# Malaria epidemiology, surveillance and response for elimination in Lao PDR

**DOI:** 10.1186/s40249-024-01202-7

**Published:** 2024-05-23

**Authors:** Chawarat Rotejanaprasert, Vilayvone Malaphone, Mayfong Mayxay, Keobouphaphone Chindavongsa, Virasack Banouvong, Boualam Khamlome, Phoutnalong Vilay, Viengxay Vanisavaeth, Richard J Maude

**Affiliations:** 1 Mahidol-Oxford Tropical Medicine Research Unit, Faculty of Tropical Medicine, Mahidol University, Bangkok, Thailand; 2 Department of Tropical Hygiene, Faculty of Tropical Medicine, Mahidol University, Bangkok, Thailand; 3 Lao-Oxford-Mahosot Hospital-Wellcome Trust Research Unit, Microbiology Laboratory, Mahosot Hospital, Vientiane, Laos; 4 Institute of Research and Education Development, University of Health Sciences, Ministry of Health, Vientiane, Laos; 5 Centre for Tropical Medicine and Global Health, Nuffield Department of Medicine, University of Oxford, Oxford, UK; 6 Center of Malariology, Parasitology, and Entomology, Vientiane, Lao PDR Laos; 7 The Open University, Milton Keynes, UK

**Keywords:** Malaria, Epidemiology, Elimination, Surveillance, Lao PDR

## Abstract

**Background:**

Lao PDR has made significant progress in malaria control. The National Strategic Plans outline ambitious targets, aiming for the elimination of *Plasmodium falciparum* and *P. vivax* malaria from all northern provinces by 2025 and national elimination by 2030. This article presents an overview of malaria epidemiology, surveillance, and response systems in Lao PDR, emphasizing experiences and achievements in transmission reduction.

**Methods:**

Data on surveillance, monitoring and evaluation systems, human resources, infrastructure, and community malaria knowledge during 2010–2020 were systematically gathered from the national program and relevant documents. The collected information was synthesized, and discussions on challenges and future prospects were provided.

**Results:**

Malaria control and elimination activities in Lao PDR were implemented at various levels, with a focus on health facility catchment areas. There has been significant progress in reducing malaria transmission throughout the country. Targeted interventions, such as case management, vector control, and community engagement, using stratification of control interventions by catchment areas have contributed to the decline in malaria cases. In elimination areas, active surveillance strategies, including case and foci investigation, are implemented to identify and stop transmission. The surveillance system has facilitated timely detection and response to malaria cases, enabling these targeted interventions in higher-risk areas.

**Conclusions:**

The malaria surveillance and response system in Lao PDR has played a crucial role in reducing transmission and advancing the country towards elimination. Challenges such as importation, drug resistance, and sustaining support require ongoing efforts. Further strengthening surveillance, improving access to services, and addressing transmission determinants are key areas of focus to achieve malaria elimination and enhance population health in Lao PDR.

**Supplementary Information:**

The online version contains supplementary material available at 10.1186/s40249-024-01202-7.

## Background

Malaria remains a significant public health challenge, affecting billions of people worldwide, particularly in tropical and subtropical regions where environmental conditions are conducive to the transmission of the disease [1, 2]. In Southeast Asia, specifically within the Greater Mekong Subregion (GMS), malaria poses a significant burden on public health and socioeconomic development [3]. The diverse epidemiology of malaria in this region is characterized by variations in disease endemicity, prevalence of different malaria species requiring distinct treatment approaches, and a wide range of vector systems with varying capacities for transmitting the parasites. Recognizing the severity of the malaria burden in the GMS, the World Health Organization (WHO) Mekong Malaria Program was launched in 1999 with the goal of reducing malaria-related morbidity and mortality, as well as containing the spread of multidrug-resistant (MDR) *Plasmodium falciparum* parasites [4, 5]. In recent years, fueled by government investments and international support, the malaria situations in GMS nations have improved greatly.

The Lao People’s Democratic Republic (Lao PDR) has made significant strides in reducing malaria incidence and is now poised to achieve elimination by 2030 [6]. The country has demonstrated a strong commitment to malaria control efforts and has benefited from favorable factors such as political commitment and evidence that appropriate interventions can effectively reduce and interrupt malaria transmission. The National Strategic Plans for malaria control and elimination, developed in five-year phases since 2016, have provided guidance for targeted interventions and received input from the Ministry of Health and scientific committees through extensive consultations and workshops involving international organizations and partners. The country aims to eliminate *P. falciparum* and *P. vivax* malaria from all northern provinces by 2025 and achieve national elimination by 2030 [7].

Building upon the successes led by the Center for Malaria Parasitology and Entomology of Lao PDR (CMPE), the planned malaria control and elimination activities aim to address current challenges and reduce the overall malaria burden in southern areas, as well as conduct elimination efforts in central and northern regions. The Ministry of Health, along with CMPE and various technical, implementation, and community partners, have intensified efforts to provide quality diagnosis and treatment services, implement effective vector control measures, and disseminate educational messages on malaria prevention and treatment-seeking behavior. Strengthening the national disease surveillance system is crucial for timely information sharing and rapid response to emerging challenges, aligning with broader health system strengthening initiatives led by the Ministry of Health. This article aimed to summarize the experiences and achievements in malaria control and elimination in Lao PDR, particularly in the elimination areas. It also discusses the challenges and prospects for scaling up these efforts nationwide. The review encompasses technical and operational aspects, highlighting gaps and needs as the country progresses toward its elimination milestones.

## Methods

### Study population

Lao PDR is a landlocked country located in Indochina, sharing borders with China, Vietnam, Cambodia, Thailand, and Myanmar. The country consists of 18 provinces, with 13 in the north and five in the south, further divided into 147 districts. The capital city, Vientiane, situated on the Mekong River along the eastern Thai border, is the largest city in the country. Lao PDR has an estimated population of 6.8 million. With its predominantly mountainous terrain, many areas in Lao PDR have elevations exceeding 500 m. Malaria in Lao PDR follows a seasonal pattern, primarily occurring during the rainy season, which lasts from June to October, characterized by the tropical monsoon climate with significant rainfall [8]. This is followed by a cooler, dry season from November to February, and a hot dry season in March and April.

### Data source and study design

Passive malaria surveillance in Lao PDR is carried out through various levels of health facilities, including village health posts, health centers, district hospitals, and provincial hospitals. Malaria cases identified in these health facilities are reported into the District Health Information System 2 (DHIS2). The DHIS2 case database allows reporting of infected individuals, capturing demographic information, village and case house locations, references to the original patient records, and diagnostic results. Data from the DHIS2 were obtained in spreadsheet format and subsequently analyzed. For this study, retrospective analysis of malaria data for the period 2010–2020 was conducted using information obtained from the Lao Ministry of Health and the National Strategic Plans. The malaria cases included in this study were confirmed through microscopy or rapid diagnostic tests.

Epidemiological trends and indicators were described to examine the incidence and proportions of different malaria species. The annual parasite incidence was calculated by dividing the total number of confirmed malaria cases by the population at risk, expressed per 1000 individuals at risk. The descriptive analyses were performed using R version 2022.07.0 software program (RStudio, PBC, Boston, MA, USA).

## Results

### Brief history of malaria epidemiology and control in Lao PDR

#### Before 2011

Malaria control efforts in Lao PDR began in 1953 with the use of the insecticide dichloro-diphenyl-trichloroethane (DDT). A centralized malaria control program was established in 1954, with DDT spraying teams deployed to all provinces, and the provision of chloroquine for malaria case management. However, due to the escalation of the Lao Civil War, spraying activities were discontinued in 1961. From 1969 to 1975, some DDT spraying took place in Vientiane province, alongside mass chloroquine administration campaigns. In 1981, the Institute of Malaria, Parasitology, and Entomology (now known as the Centre of Malariology, Parasitology, and Entomology - CMPE) was established, along with a nationwide network of malaria units. In 1988, insecticide-treated bednets (ITNs) were introduced as a primary control measure, which were then scaled-up nationally with support from the World Bank and Asian Development Bank during the mid-1990s.

Additional support with ITNs was provided by the Laos-Europe Malaria Control Program from 1997 to 2001. By the early 2000s, the dynamics of malaria transmission in Lao PDR were changing, largely due to the implementation of large-scale malaria control projects that focused on the distribution of ITNs and the use of artemisinin-based combination therapies (ACTs). Malaria posed a significant threat to the entire country, accounting for a substantial portion of morbidity and mortality, with 70% of the population at risk in 2003. Nonetheless, control and elimination efforts sought to diminish the malaria burden, aiming for a more localized transmission pattern in the northern and central provinces [9]. Over the period 2000 to 2010, the number of probable and confirmed cases fell by 92%, from 279,903 to 23,047 and the number of malaria related deaths decreased from 350 to 24. To formalize and implement malaria control policies, the National Strategic Plan for Malaria Control and Elimination (NSPMCE) for 2011–2015 was finalized in 2009, serving as a 5-year strategy for malaria control and pre-elimination. The development of these policies involved the Ministry of Health, the scientific committee, and various stakeholders at different levels, including international organizations and partners.

### During 2011–2015

Malaria incidence in Lao PDR declined in the northern regions until 2011. However, from December 2011, outbreaks in the southern areas caused a significant rise in confirmed cases. In 2012, there were 46,202 reported cases, increasing to 50,674 by 2014 nationwide. Individuals in these regions, less familiar with malaria threats, might have been more susceptible to severe cases or death due to lack of immunity. The majority of reported cases in Lao PDR from 2010 to 2020 were in the southern provinces. The proportion of confirmed male cases increased significantly from 46% in 2009 to 86% in 2014, suggesting a correlation between susceptibility and specific high-risk occupations.

Starting in 2011, there was a malaria outbreak in the southern provinces of Lao PDR, reaching its peak in December 2012 and continuing through 2014. This epidemic was likely influenced by changes in population movements due to increased economic activities such as unregulated deforestation and the implementation of large-scale development projects including hydropower dams, mining operations, and plantations. Climatic conditions, including rainfall patterns, temperature, and humidity, also played a role. While malaria-related deaths had decreased from 350 in 2000 to just 17 in 2011, the number of recorded deaths resurged due to outbreaks in the southern provinces, reaching 44 in 2012 followed by 28 in 2013 and 4 in 2014. Malaria mortality was highest in the southern region, where malaria incidence was also the highest.

**Fig. 1:**
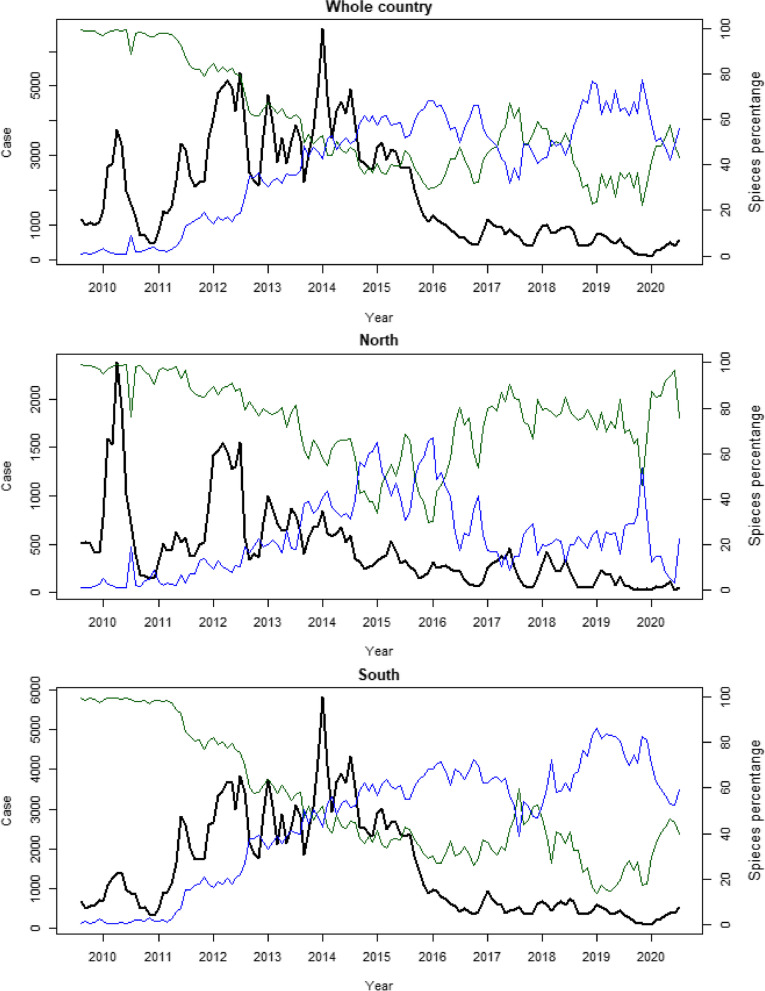
Plots of total monthly positive cases (black) with percentages of *P. falciparum* (green) and *P. vivax* cases (blue) for the whole country (top), north (middle) and south (bottom) during 2010–2020

**Fig. 2:**
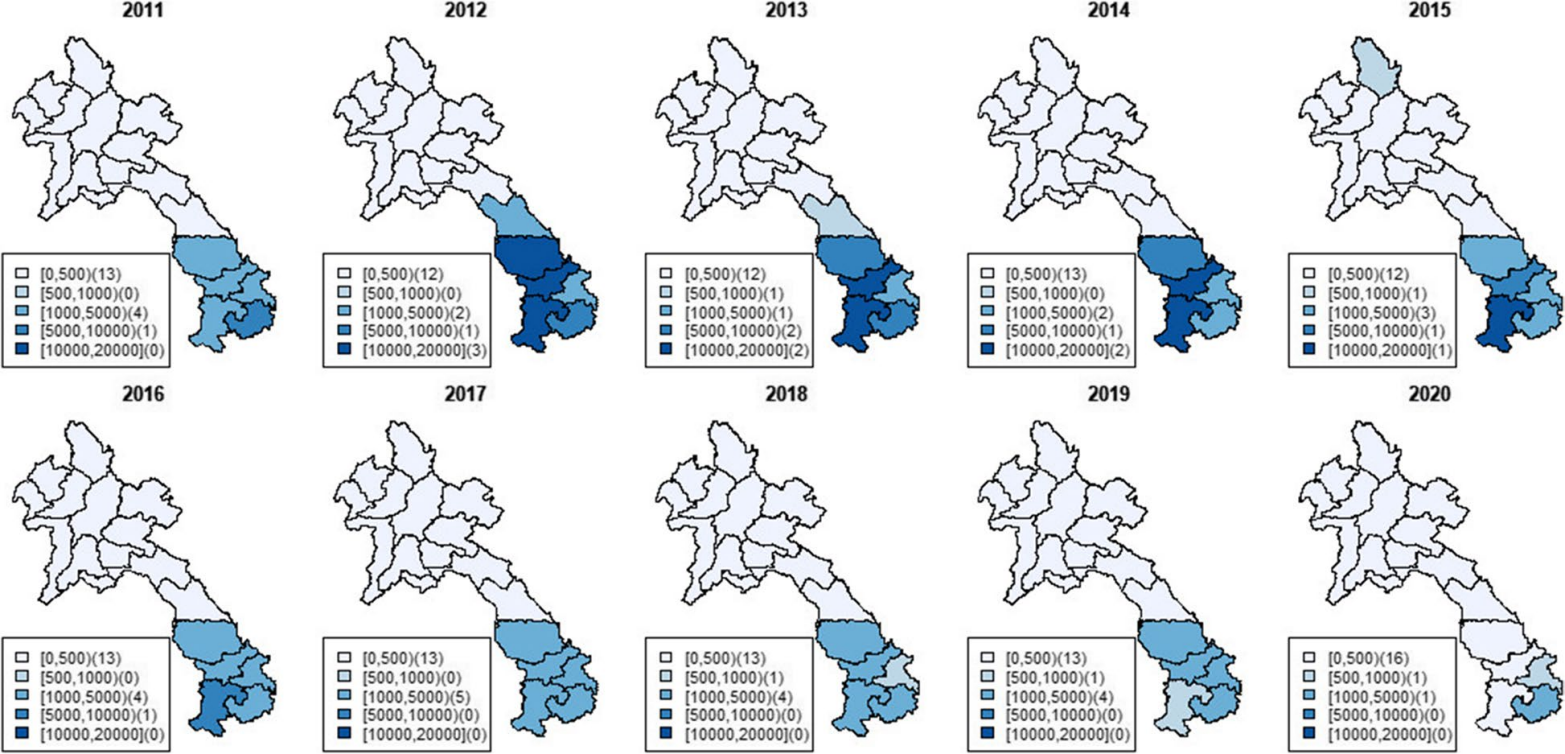
Maps of provincial total malaria incidence per 100,000 population in Lao PDR during 2011–2020

Following the implementation of the National Strategic Plan for Malaria Control and Pre-Elimination (2011*–*2015), an initial version of the malaria National Strategic Plan (NSP) for 2016–2021 was developed in 2014. The NSP aligned with regional objectives and activities aimed at controlling malaria and preventing the emergence of artemisinin resistance. To achieve the goal of malaria elimination by 2030, the 2016*–*2020 NSP represented the first phase of a three-phase approach to eliminate all forms of malaria in Lao PDR. The plan focused on strengthening interventions in the southern part of the country to reduce the primary malaria burden, while also initiating efforts to eliminate malaria in the remaining focal areas in central and northern Lao PDR.

Over the past decade, Lao PDR has observed a significant reduction in malaria incidence. The number of malaria cases decreased from 279,903 cases in 2001 to 6,409 cases in 2019. The introduction of rapid diagnostic tests (RDTs) in 2012 initially led to a rise in reported malaria incidence [7]. However, in recent years, substantial progress has been achieved in reducing malaria in Lao PDR through various large-scale control projects and support, notably funded by the Global Fund to Fight AIDS, TB, and Malaria. The substantial impact of increased ITN coverage, quality ACT distribution, and the rapid rollout of diagnostics is evident, particularly in reducing transmission in the northern and central provinces. Though challenges persist in the southern provinces due to forest-going behavior and agricultural practices, this control and elimination progress highlights effective program management, and consistent government and partner support.

### From 2016–present

Though the overall trend of malaria in Lao PDR has been downward since 2014 (Figs. 1 and 2), the disease continues to be a significant public health concern, and the country remains at risk of epidemics. In response, the Ministry of Health, along with the Department of Communicable Disease Control and the Centre for Malariology, Parasitology and Entomology (CMPE), in collaboration with the WHO and other technical partners, developed a new NSP in 2020. The current NSP, released in December 2020, aims to be implemented from 2021 to 2025 as the second phase of a three-phase approach toward eliminating all forms of malaria in Lao PDR and preparing the country for national elimination by 2030. Its objectives include strengthening interventions focused on reducing the primary malaria burden in the southern region and expanding efforts to eliminate malaria in low-burden focal areas across the entire country. A key component of this new plan is using data to stratify the country to target resources for control and elimination, an initiative which began in 2019, as described below.

### Malaria stratification and control interventions

Malaria stratification is a crucial process for targeting interventions in specific geographic areas to effectively interrupt malaria transmission. As Lao PDR progresses from burden reduction to elimination, it is essential to tailor interventions to village foci or health facility catchment areas (HFCAs). This approach ensures that resources are appropriately directed based on case history, receptivity, and vulnerability, leading to the interruption of malaria transmission [10]. Lao PDR has successfully established a sensitive surveillance system, providing monthly updates through DHIS2, which collects passive case data from public health facilities, private health vendors, and community health workers. The availability and comprehensiveness of these surveillance data enable fine-scale mapping down to the level of health facility catchment areas. The goal of malaria stratification is to analyze the latest epidemiological data to support the disease program in planning for the 2021*–*2025 strategic plan, including the planning and costing of malaria commodities such as diagnostics, treatment, prevention, and the expansion of service availability points through the village health volunteer (VHV) and village malaria worker (VMW) networks.

The stratification exercise conducted in 2019 involved a two-step process. Firstly, at the district level, stratification was based on the annual parasite incidence (API) using data from January 2017 to September 2019. This determined whether districts should focus on burden reduction activities (API > 1) or elimination activities (API < 1) (Table 1). Secondly, at the health facility catchment area level, a caseload-based stratification was performed to identify HFCAs within each district that required intensified intervention packages. A risk map, incorporating demographic, environmental, and ecological data, was used alongside the case data for complementation and validation. HFCAs were classified into four strata based on caseload over the previous two years and nine months: Stratum 1 (malaria-free), Stratum 2 (low risk, < 5 cases), Stratum 3 (moderate risk, 5–20 cases), and Stratum 4 (high risk, > 20 cases). The 2019 stratification exercise revealed that out of Lao PDR’s 148 districts, 125 should focus on elimination activities, while the remaining 23 should focus on burden reduction (Table 1).

Moving forward, as Lao PDR advances towards elimination, finer scale mapping and more specific stratification at the health facility catchment area level are required. Regular stratification exercises are planned throughout the current NSP implementation period. In 2022, another stratification exercise was conducted, resulting in an increase in the number of malaria-free HFCAs. The total number of HFCAs in strata 2 and 3 remained similarly (Table 2), but their geographical distribution has changed. Notably, there has been a significant decrease in the number of HFCAs classified under stratum 4. Furthermore, the total population in HFCAs in strata 2, 3, and 4, has reduced by 27% when considering the estimated population for 2022. At the district level, the stratification process also revealed a transition. In 2019, there were 125 districts classified as malaria elimination areas and 23 districts under burden reduction, while in 2022, the numbers shifted to 134 districts classified as malaria elimination areas and 14 as burden reduction districts, marking a nine-district reduction in the burden reduction category compared to the status in 2019. These stratification exercises and population changes provide valuable insights for the malaria program, enabling more targeted and effective interventions to achieve the goal of malaria elimination in Lao PDR.

**Table 1:** District level API-based stratification of malaria risk in 2019 in Lao PDR

Province	Number of districts	Elimination API < 1	Burden reduction API > 1
Vientiane C.	9	9 (100%)	0
Phongsaly	7	7 (100%)	0
Luangnamtha	5	5 (100%)	0
Oudomxay	7	7 (100%)	0
Bokeo	5	5 (100%)	0
Luangpraband	12	12 (100%)	0
Huaphanh	10	10 (100%)	0
Xaybury	11	11 (100%)	0
Xiengkhuang	7	7 (100%)	0
Vientiane. P	11	11 (100%)	0
Borikhamxay	7	7 (100%)	0
Khammuane	10	10 (100%)	0
Savannakhet	15	11 (73.3%)	4 (27.7%)
Saravane	8	1 (12.5%)	7 (87.5%)
Sekong	4	1 (25%)	3 (75%)
Champassack	10	6 (60%)	4 (40%)
Attapeu	5	0	5 (100%)
Xaysomboun	5	5 (100%)	0
**Total**	**148**	**125 (84%)**	**23 (16%)**

*API *Annual parasite incidence

The results of the stratification exercise played a crucial role in guiding the allocation of interventions and estimating the costs for the NSP 2021–2025. Recognizing the dynamic nature of malaria transmission patterns, the program continually adapts its allocations. Although universal coverage of malaria testing and treatment is ensured across all HFCAs regardless of risk, the minimum stock for these services may vary based on local transmission risk. Information, education, and communication (IEC) materials are universally accessible in all HFCAs. Those identified as malaria-free or of very low risk exclusively receive these two essential interventions — universal testing and treatment, and IEC. Refer to Table 3 for a comprehensive breakdown of specific interventions tailored to each HFCA stratum.

**Table 2:** Health facility catchment area level caseload-based stratification of malaria risk

Stratification classification	2019 Stratification		2022 Stratification		Change 2019 vs. 2022
	No. HFCA	2022 population estimate	No. HFCA	2022 population estimate	
**Stratum 1 (Malaria free)**	752	4,161,390	849	5,034,669	+ 97 HFCA + 900,000 population
**Stratum 2 (Low risk)**	196	1,464,610	193	1,243,685	No change
**Stratum 3 (Medium risk)**	96	555,283	97	638,777	
**Stratum 4 (High risk)**	185	1,199,753	88	483,606	− 97 HFCA − 700,000 population
**Grand Total**	1229	7,381,036	1227	7,400,737	

*HFCA *Health Facility Catchment area

Moderate and high-risk strata will receive additional interventions beyond universal access to quality testing, treatment, and IEC activities. This includes the provision of VHVs/VMWs and long-lasting insecticidal nets (LLINs) in targeted high-burden villages within the moderate-risk stratum, as well as outbreak response in burden reduction areas and foci response in elimination areas. In the highest-risk areas (stratum 4), all villages in the catchment area will be allocated VHVs and will be eligible to receive an LLIN every 3 years. Case-based surveillance and implementation of related elimination response activities, such as notification, investigation, classification, and foci response, will also be applied to all strata within elimination districts.

**Table 3:** Intervention packages for HFCA strata

Strata	Intervention packages	Case based surveillance and response are applied as an intervention in all elimination districts
Stratum 1: Malaria Free	1. Universal testing and treatment 2. IEC	
Stratum 2: Low risk	1. Universal testing and treatment 2. IEC	
Stratum 3: Moderate risk	1. Universal testing and treatment 2. Universal LLIN coverage 3. Targeted VMWs (villages with population at risk) 4. Outbreak or foci response (ACD, IRS, entomological surveillance, LLIN) 5. IEC	
Stratum 4: High risk	1. Universal testing and treatment 2. Universal LLIN coverage 3. Targeted VMWs (including FTAT) 4. Outbreak or foci response (ACD, IRS, entomological surveillance, LLIN) 5. IEC	

*ACD* Active Case Detection, *IRS* Indoor Residual Spraying, *LLIN* Long-Lasting Insecticide-treated Net, *IEC* Information Education and Communication, *HFCA* Health Facility Catchment area

### Malaria surveillance and response in elimination areas

The NSP for malaria control and elimination 2021–2025 focuses on eliminating *P. falciparum* from the entire country and all forms of malaria from thirteen northern provinces. The aim is to reduce the annual incidence of *P. vivax* in the five southern provinces to less than 1 case per 1,000 population. *P. falciparum* in the thirteen northern provinces was targeted for elimination by 2021. In elimination areas, active surveillance strategies, including case and foci investigation, are implemented to identify and stop transmission.

Case-based surveillance has been implemented in elimination areas due to reduced transmission levels and variations in malaria case distribution. A district is now considered an elimination district if it achieves an annual incidence rate of less than 1 case per 1000 population, supported by factors such as a trained workforce, allocated rapid response budgets, adequate transportation, necessary supplies, and a suitable data management system capable of conducting surveillance activities in elimination areas. These considerations, encompassing case detection, notification, investigation, and response, collectively inform the decision to transition a district into the elimination phase. This approach ensures seamless coordination between the national malaria control program and the health system.

In areas with low malaria transmission, surveillance plays a crucial role beyond routine data collection. The objectives of the surveillance system in these areas are to detect all malaria infections through quality-assured diagnostic tests, treat and cure infections with appropriate treatment, notify positive cases within 24 h, and ensure accurate recording of cases. Investigations are conducted to classify cases accordingly as in Table 4. The scaling up of elimination in Lao PDR is now part of the re-stratification process conducted every two years. Districts with transmission levels below 1 per 1000 API and the necessary surveillance and response structures and resources for case-based investigations are adopting elimination activities. Annual assessments and targeted support from relevant stakeholders are conducted to review impact, effectiveness, potential improvements, and the suitability of nationwide scale-up for all districts entering the elimination phase.

**Table 4:** Malaria case classification

Case classification	Epidemiological significance
1. Relapse/recrudescence	Not locally acquired
2. Induced	Not locally acquired
3. Imported from another District	Not locally acquired
4. Imported from another Province	Not locally acquired
5. Imported from another Country	Not locally acquired
6. Introduced	Locally acquired
7. Indigenous	Locally acquired

### Malaria surveillance system

The national malaria control program began implementing malaria elimination activities in mid-2018, focusing on timely notification, investigation, classification, and response for all malaria cases detected within elimination areas. In collaboration with WHO and other technical partners, the Ministry of Health, particularly the Department of Communicable Disease Control and the Centre for Malariology, Parasitology, and Entomology (CMPE), led the development of the new national malaria strategic plan in 2020. This initiative involved an inclusive process, encompassing a comprehensive malaria program review in 2019. The review assessed the challenges and successes of implementing the 1*–*3*–*7 elimination strategy introduced in 2018, providing valuable recommendations for enhancing and incorporating strategies into the National Malaria Strategic Plan for 2021–2025. When malaria burden reduces to API < 1 per 1000, surveillance and response efforts should be intensified. Following the 1*–*3*–*7 strategy, confirmed cases are notified within one day by various health facilities, including community-based workers, private sector, and hospitals, to district and provincial anti-malaria units. Case investigations are conducted at the point of care, and remote support is provided by District Anti-Malaria Nuclei (DAMN) or Provincial Anti-Malaria Station (PAMS) units for classification protocols.

Within three days of diagnosis, PAMS or DAMN verifies case investigation results and conducts active case detection if the case is imported. For locally acquired cases, a foci response is initiated within seven days, involving broader activities. If an incidence is classified as a local infection (indigenous or introduced cases), district and/or provincial malaria officials should initiate a comprehensive response. This may involve conducting active case detection (ACD) blood tests, implementing vector control measures such as LLINs and/or IRS, and providing community health education. Cases are classified based on origin, including indigenous, introduced, imported, induced, recrudescence, or relapse. To improve efficiency, the program plans to adopt a flexible and pragmatic approach to the 1*-*3*-*7 strategy, with case investigations and classifications conducted by health facility staff. Immediate communication is established with elimination focal points for locally acquired cases, leading to focus investigations and response within seven days. Focus investigations include reactive case detection (RACD) among household members and peers of the patient, and the response is tailored to local circumstances.

### Case detection

**Fig. 3:**
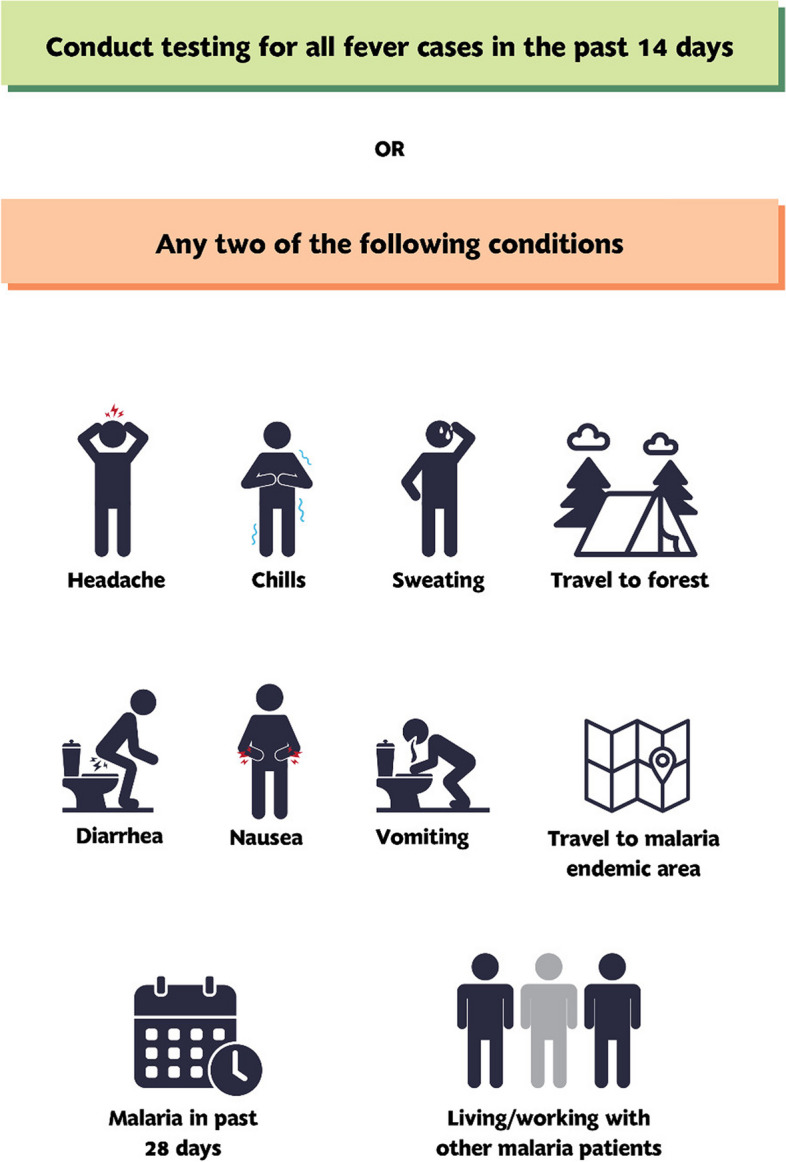
Criteria for testing of suspected malaria cases in PCD (as per the NMTG)

Case detection is a crucial component of surveillance operations aimed at identifying malaria infections in the community. It involves screening symptomatic individuals using diagnostic tests such as RDT or microscopy. Two approaches are commonly used for case detection: passive case detection (PCD) and active case detection (ACD). PCD focuses on detecting malaria cases among individuals who voluntarily visit a healthcare facility or healthcare provider seeking treatment, particularly for febrile illness. In Lao PDR, PCD is the primary method of malaria case detection and is carried out across various levels of healthcare, including hospitals, health centers (HC), village health workers (VHW), malaria posts, and the public-private mix (PPM) [11–13]. The national malaria treatment guidelines (NMTG) and PCD Standard Operating Procedures provide criteria for testing suspected malaria cases. In these, all patients presenting with a fever in the past 14 days or with any two of a list of symptoms and risk factors should be tested for malaria (Fig. 3). It is also important to consider travel history as a criterion to assess potential malaria infection and exclude indigenous transmission, especially in elimination settings. As part of the case investigation process, travel history within the past two weeks will be collected, including information on visited locations.

ACD involves health workers actively detecting malaria cases at the community or household level, focusing on population groups considered at high risk. ACD can be conducted through fever screening followed by parasitological examination of all febrile patients or by directly performing parasitological examination in target populations without prior fever screening. ACD plays a critical role in complementing PCD and detecting both symptomatic and asymptomatic malaria infections at an early stage. It enables prompt and effective treatment and immediate response to prevent secondary cases. There are two types of ACD: RACD and proactive case detection (PACD). RACD is conducted in response to confirmed cases or clusters of cases, screening and testing populations potentially linked to these cases. PACD, on the other hand, may be carried out in high-risk groups without being prompted by the detection of cases.

### Case notification

**Fig. 4:**
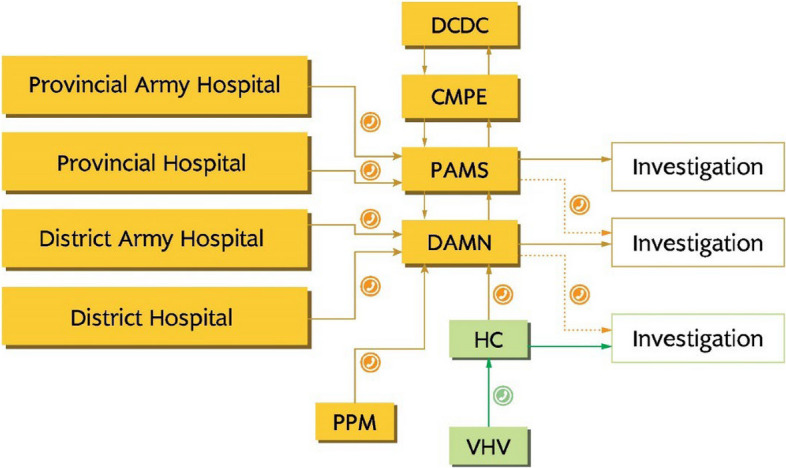
Communication flow after case detection to initiate investigation. DPCDC: District and Provincial Communicable Disease Control, CMPE: Center for Malaria Parasitology and Entomology, PAMS: Provincial Anti-Malaria Station, DAMN: District Anti-Malaria Nuclei, HC: Health Center, PPM: Public-Private Mix, VHV: Village Health Volunteer

All confirmed malaria cases diagnosed at public sector health facilities, private facilities, including health centers, district or military hospitals, and public-private mix sites, must be notified to the DAMN within one day of diagnosis through a phone call. Similarly, cases diagnosed at public Provincial Hospitals or Provincial Military/Police Hospitals should be notified to the PAMS via a phone call. For cases diagnosed at the community level by VHVs, they should be notified within one day by phone call to the nearest HC, which will then inform the DAMN. Figure 4 provides a visual representation of the malaria case notification communication process and the subsequent flowchart for case investigation.

### Case and foci investigation and response

In settings of malaria elimination, case and foci investigation play a crucial role in gathering information about confirmed malaria cases, including patient details, travel history, and response interventions. In Lao PDR, it is required to investigate each notified case of confirmed malaria within three days of diagnosis. This investigation is conducted by health center staff, PAMS, DAMN, or VHV, depending on the catchment area in which the case is detected. The case investigation aims to classify the case based on the origin of infection, especially to determine if the patient acquired the disease locally, indicating ongoing local transmission or other factors that may contribute to onward transmission. The investigation includes the following components:

- Case details: Gathering patient demographic and contact information, as well as clinical details of the current infection such as date of onset, species, and treatment.
- Patient history: Obtaining the patient’s travel history (domestic and international) over the past two weeks, previous malaria history, blood transfusion history, family members’ malaria history, individual and household bed net status, and history of travel to forests.
- Case classification: Based on the responses from the case investigation, particularly clinical case details and patient travel history, the case is classified accordingly as either local or imported.
- Household and travel companion testing (for non-locally acquired cases only): Documenting details of RACD conducted in high-risk populations to identify potential onward transmission.

Malaria case investigation teams typically consist of the health center staff responsible for diagnosing the case. In cases detected by district or provincial hospitals, DAMN or PAMS, respectively, should be involved. As the capacity of health center staff increases for case classification, VHVs who provide point-of-care malaria services may also assist by conducting simplified case classification on the first day under the guidance of health center staff. A case investigation form should be completed for each confirmed malaria case, and an electronic copy of the completed case notification, classification, and response information should be sent to DAMN for entry in DHIS2 by the fourth day after diagnosis. The original paper form should be forwarded to DAMN for filing, future reference, and verification purposes (after day 90) once the follow-up and integrated Drug Efficacy Surveillance (iDES) section are completed.

**Fig. 5:**
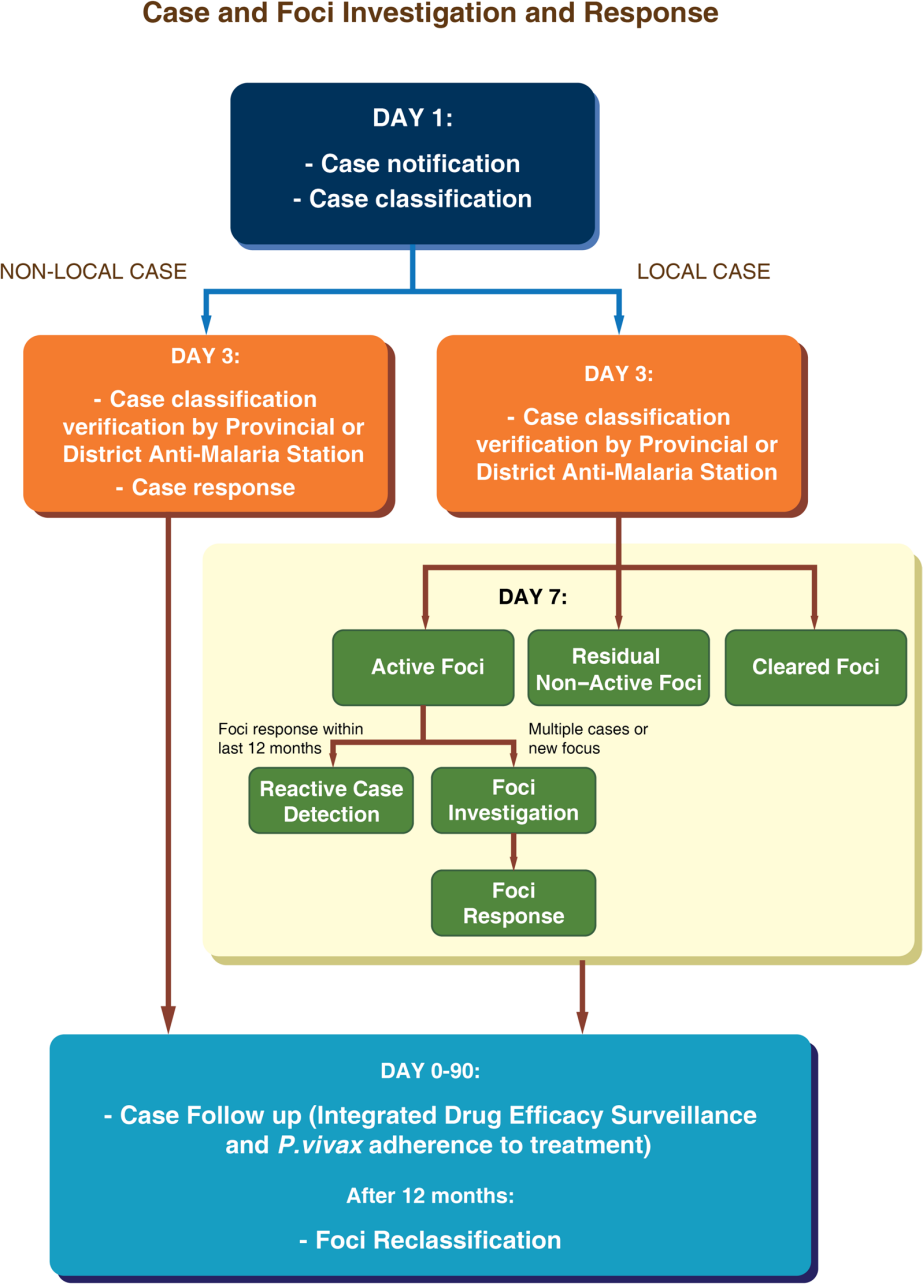
Case and foci classification and response process

A malaria focus refers to a village situated in a currently or formerly receptive endemic area with the necessary epidemiological and ecological factors for malaria transmission, either continuously or intermittently. Upon detecting a case of locally acquired malaria, a focus investigation should usually be conducted within seven days of case diagnosis. However, if available data suggests that the case is from an active focus that has already been investigated and responded to within the previous 12 months, another focus investigation may not be necessary. Figure 5 illustrates the flowchart detailing the case and foci classification and the corresponding response process.

In areas with transmission foci, the staff of CMPE will conduct entomological assessments to identify any issues that may undermine malaria control efforts, such as insecticide resistance or changes in vector behavior. As part of the routine annual response in residual non-active foci, PACD can also be carried out. PACD involves screening populations based on symptoms and criteria outlined in the NMTGs. To ensure effective implementation of surveillance protocols, notification and case and foci investigation training are provided to all relevant stakeholders, including Primary Case Detection and Classification (PCDC) teams, provincial and district malaria officers, HCs, VHVs, PPM facilities, hospitals, and military personnel. Training is also extended to Rapid Response Team (RRT) staff to equip them with the necessary skills for responding to notifiable diseases, including malaria. Furthermore, it is essential to include malaria staff in District and Provincial Communicable Disease Control (DPCDC) training, along with the field epidemiology training staff from the National Center for Laboratory and Epidemiology (NCLE), as this integration will facilitate coordinated efforts across all communicable diseases.

### Human and financial resources

Human and financial resources pose significant challenges to malaria elimination in Lao PDR. The limited availability of a skilled workforce, including healthcare workers, entomologists, and epidemiologists, hampers the effective implementation of elimination strategies. Shortages of field staff, especially in remote areas, constrain interventions’ reach. Additionally, providing adequate training for healthcare professionals and community workers involved in malaria control remains a challenge.

Despite financial constraints, it is notable that over half of the funding for malaria programs in Lao PDR originates from international partners, highlighting a significant global commitment. Ensuring the sustainability of this funding amidst resource limitations and competition with other health priorities is crucial. To mobilize domestic resources, CMPE aims to strengthen collaboration with the Ministry of Finance and Parliament, exploring avenues to secure adequate funding for civil servant salaries and infrastructure. Relevant public health authorities plan to underscore transparency, accountability, and efficiency in resource disbursement to facilitate effective collaboration with the government and financial partners. Hence, collaboration with international partners holds the potential to develop innovative strategies and secure resources.

Addressing these human and financial challenges is vital for malaria elimination. It necessitates joint efforts from domestic and international stakeholders to ensure a skilled workforce, sufficient funding, and equitable resource distribution. Long-term commitments, capacity-building, and innovative financing mechanisms are essential for substantial progress towards malaria elimination in the country.

## Discussion

Lao PDR has made significant progress in controlling malaria. The country has demonstrated a strong commitment to malaria control efforts and has achieved notable successes in reducing malaria incidence and related mortality. However, to achieve and sustain malaria elimination in Lao PDR, several key challenges need to be addressed. One is the geographic and ecological diversity of the country, which contributes to varying levels of malaria transmission intensity across different regions. Remote and hard-to-reach areas, particularly along the borders and in mountainous regions, pose logistical difficulties in accessing communities and delivering effective malaria interventions. Another challenge is the presence of mobile and migrant populations, including cross-border movements and seasonal workers. In a community-based survey conducted in four districts of Savannakhet Province from February to June 2015 [14], the majority of migrant workers were male (75.7%) and Vietnamese (92.6%). The median age (interquartile range) was 31 (25 to 41) years. The common occupations were factory worker (47.6%), trader/shopkeeper (21.5%), and farmer (16.4%).

Reaching populations in mobile communities with consistent malaria prevention and treatment services presents a challenge, impeding the interruption of transmission. It is crucial to engage and implement targeted interventions for these populations, possibly through mobile clinics or outreach services, to enhance effective malaria control and elimination. In Lao PDR’s comprehensive service delivery model, public facilities and a network of VMWs play a pivotal role. Supported by external assistance and VHVs, VMWs contribute significantly to delivering early diagnosis and treatment to remote malaria-endemic areas. CMPE is dedicated to sustaining the program’s strength, ensuring VMWs’ community standing, and providing necessary supervision, commodities, and remuneration.

Furthermore, the presence of drug-resistant *P. falciparum* poses a significant challenge to elimination efforts [15–17]. The emergence and dissemination of antimalarial drug resistance can compromise treatment effectiveness and elevate the risk of treatment failure. Regular monitoring of drug resistance patterns and prompt adjustment of treatment strategies are imperative to curb the further spread of drug-resistant parasites. A decade ago, artemisinin resistance surfaced in *P. falciparum* in Cambodia, instigating extensive research across the GMS [18]. The epicenter of resistance lies in the Eastern GMS, encompassing Cambodia, Vietnam, Lao PDR, and Eastern Thailand [16, 19]. Evaluation of triple artemisinin combination therapies has been pursued to address this concern [18]. The GMS, characterized by substantial population movement across borders, facilitates malaria transmission and cross-border introduction [20]. Research at the China–Myanmar border reveals evidence of malaria importation along international borders [21, 22]. Establishing a comprehensive disease and vector surveillance system at sentinel sites in border areas of neighboring countries allows timely detection and management of malaria cases, furnishing updated knowledge for effective control measures and facilitating efficacy studies of antimalarials in the GMS.

The use of traditional vector control methods such as LLINs and IRS has been instrumental in reducing malaria burden [23]. However, challenges persist with the effectiveness of these methods against *Aedes* mosquitoes, particularly due to insecticide resistance and operational limitations [24]. To address these challenges, the program has explored vector control strategies tailored to the diverse epidemiological profiles and behaviors of vectors in Lao PDR. This includes expanding the roles of VMWs, targeted testing in high-risk populations, deploying mobile malaria workers along key travel routes, and providing quality case management for migrant workers. Additionally, the program has considered the procurement and distribution of forest packs and repellents, and exploring the use of new vector control tools such as topical repellent [25].

Strengthening surveillance systems is a critical challenge [26, 27]. This includes improving the capacity for accurate and timely case detection, reporting, and data management. In remote and resource-limited settings, the availability and accessibility of diagnostic tools and trained personnel for accurate diagnosis may be limited. Enhancing the diagnostic capacity and ensuring the quality of surveillance data are essential for effective targeting of interventions and monitoring of progress. While persistent challenges in financial and human resource availability highlight the necessity for sustainable funding to support comprehensive malaria control and elimination strategies, it is crucial to emphasize that building and maintaining a skilled workforce, including healthcare professionals and community health workers, is essential for effective program implementation and long-term sustainability [28, 29].

Addressing these challenges requires a multi-sectoral and integrated approach, with strong leadership and coordination among various stakeholders, including the government, non-governmental organizations, development partners, and local communities [30, 31]. It is essential to invest in strengthening health systems, improving access to quality healthcare services, and promoting community engagement to overcome the barriers to malaria elimination in Laos [32, 33]. In addition to the challenges, it is important to acknowledge that the government, in collaboration with development partners and international organizations, has played a key role in driving these efforts. Their commitment to providing resources, technical support, and policy guidance has contributed to the successful implementation of malaria control programs. The achievements in malaria control in Lao PDR demonstrate that with effective partnerships and sustained investments, it is possible to make significant strides towards eliminating malaria. The country’s success serves as a testament to the dedication and hard work of healthcare professionals, researchers, community health workers, and individuals at all levels who have contributed to the fight against malaria. While challenges remain, the progress made thus far provides a solid foundation for further advancing malaria elimination efforts in Lao PDR. By building upon existing successes, addressing the remaining barriers, and leveraging the lessons learned, the country can continue its journey towards a malaria-free future.

## Conclusions

While challenges persist in the path toward malaria elimination in Lao PDR, the Ministry of Health stresses the importance of sustaining momentum for a malaria-free environment. Challenges in human resources, financial systems, and capacity building at sub-national levels are crucial considerations in the national malaria strategic plan. Opportunities exist in adopting health reform strategies, especially in strengthening sub-national capacity and enhancing primary health care delivery to underserved populations. Notable progress in malaria control and elimination has been made through targeted interventions, robust surveillance, and community engagement. However, challenges, including importation, maintaining support, and drug resistance, persist, requiring ongoing efforts. Key focus areas for the future include strengthening surveillance, improving service accessibility, and addressing transmission determinants, with lessons learned applicable to other regions. Continued commitment and collaboration are essential for achieving malaria elimination and enhancing population health in Lao PDR.

### Supplementary Information

Supplementary Material 1.

## Data Availability

The data that support the findings of this study were obtained from Center of Malariology, Parasitology, and Entomology, Vientiane, Lao PDR, but restrictions apply to the availability of these data, which were used with permission for the current study, and are therefore not publicly available.

## Abbreviations

CMPE: Center for Malaria Parasitology and Entomology of Lao PDR
DHIS2: District Health Information System 2
NSP: National Strategic Plan
NMTG: National Malaria Treatment Guidelines
HFCA: Health Facility Catchment Area
VHV: Village Health Volunteer
VMW: Village Malaria Worker
ACD: Active Case Detection
IRS: Indoor Residual Spraying
LLIN: Long-Lasting Insecticide-treated Net
IEC: Information, Education and Communication
DAMN: District Anti-Malaria Nuclei
PAMS: Provincial Anti-Malaria Station
iDES: integrated Drug Efficacy Surveillance
