# Correction: Malaria epidemiology, surveillance and response for elimination in Lao PDR

**DOI:** 10.1186/s40249-024-01210-7

**Published:** 2024-05-31

**Authors:** Chawarat Rotejanaprasert, Vilayvone Malaphone, Mayfong Mayxay, Keobouphaphone Chindavongsa, Virasack Banouvong, Boualam Khamlome, Phoutnalong Vilay, Viengxay Vanisavaeth, Richard J. Maude

**Affiliations:** 1 Mahidol-Oxford Tropical Medicine Research Unit, Faculty of Tropical Medicine, Mahidol University, Bangkok, Thailand; 2 Department of Tropical Hygiene, Faculty of Tropical Medicine, Mahidol University, Bangkok, Thailand; 3 Lao-Oxford-Mahosot Hospital-Wellcome Trust Research Unit, Microbiology Laboratory, Mahosot Hospital, Vientiane, Laos; 4 Institute of Research and Education Development, University of Health Sciences, Ministry of Health, Vientiane, Laos; 5 Centre for Tropical Medicine and Global Health, Nuffield Department of Medicine, University of Oxford, Oxford, UK; 6 Center of Malariology, Parasitology, and Entomology, Vientiane, Lao PDR Laos; 7 The Open University, Milton Keynes, UK

**Correction: Infect Dis Poverty 13, 35 (2024)**

**https://doi.org/10.1186/s40249-024-01202-7**

Following publication of the original article [1], the authors reported a typo in one of the author names.

The name “Mayfong Maysay” should be corrected to “Mayfong Mayxay”.

The original article [1] has been updated.
