# Supplementary material for: Malaria epidemiology, surveillance and response for elimination in Lao PDR
Source: Infect Dis Poverty. 2024 May 23;13:35. doi: 10.1186/s40249-024-01202-7 (PMC11112833; doi:10.1186/s40249-024-01202-7)
Supplement: Supplementary file 1 — Supplementary Material 1. [file 40249_2024_1202_MOESM1_ESM.docx]

Dear Editor and Reviewer,

We sincerely appreciate the opportunity to revise our manuscript. We believe that the manuscript has significantly benefited from these recommendations. Please find our response to the editor and reviewer comments below in italics, marked with track changes in the revised manuscript. We are grateful for your consideration.

Best regards,

Chawarat Rotejanaprasert

Reviewer #4: The team has done a great job summarising the progresses made so far and efforts aimed at controlling and trying to achieve elimination in Lao PDR . There are so many lessons other malaria programs can learn from the Lao PDR from surveillance, sub-national stratification, to fine scale stratification, targeted intervention, community involvement, national government investment to malaria program, external and partner coordinated effort and support etc….and how such decisions can accelerate reduction in malaria.

While the team has described well how they respond to cases, it's not clear how vector control responses is performed and what control measures are implemented in cases of vector control response to outbreaks.

- *Thank you for your comments. We have included details on vector control measures in the malaria surveillance system section. We outlined that if an incidence is classified as a local infection (indigenous or introduced cases), district and/or provincial malaria officials should initiate a comprehensive response, which may include conducting active case detection (ACD) blood tests, implementing vector control measures such as LLINs and/or IRS, and providing community health education.*

It would be great to detail some of the vector measures deployed outside ITNs and IRS? While they have lightly touched on climate/weather implication in malaria control and elimination efforts, its not clear whether their analysis has integrated climate or weather patterns in each geographical setting.

- *Thank you for your feedback. We have addressed your concern by adding a paragraph discussing vector control in Lao PDR. As for integrating climate data, we conducted analysis on space-time patterns and their association with weather, however we have chosen to present these findings in a separate paper, which has been submitted to a journal.*

Editor comment

We have edited the manuscript according to the journal’s requirement, please see the edited version in attachment. But there still are some minor issues to be addressed or revised, and the comment/guidance has been noted in the attached version. Please confirm the edits and modify the manuscript in the attached track-change version, and provide the point-to-point response.
Please ask all authors carefully confirm the latest version.

- *Thank you for your feedback. We have addressed the editor's comments point-by-point. The attached file has been updated to reflect these changes, and we have also incorporated the suggestions made by reviewer 4, along with the editor's comments, in the revised version with track changes.*
